# Frameworks for Preventing, Detecting, and Controlling Zoonotic Diseases

**DOI:** 10.3201/eid2313.170601

**Published:** 2017-12

**Authors:** Miriam L. Shiferaw, Jeffrey B. Doty, Giorgi Maghlakelidze, Juliette Morgan, Ekaterine Khmaladze, Otar Parkadze, Marina Donduashvili, Emile Okitolonda Wemakoy, Jean-Jacques Muyembe, Leopold Mulumba, Jean Malekani, Joelle Kabamba, Theresa Kanter, Linda Lucy Boulanger, Abraham Haile, Abyot Bekele, Meseret Bekele, Kasahun Tafese, Andrea A. McCollum, Mary G. Reynolds

**Affiliations:** US Centers for Disease Control and Prevention, Atlanta, Georgia, USA (M.L. Shiferaw, J.B. Doty, J. Morgan, T. Kanter, L.L. Boulanger, A.A. McCollum, M.G. Reynolds);; US Centers for Disease Control and Prevention, Tbilisi, Georgia (G. Maghlakelidze, J. Morgan);; National Center for Disease Control and Public Health, Tbilisi (E. Khmaladze);; National Food Agency, Tbilisi (O. Parkadze);; Laboratory of the Ministry of Agriculture, Tbilisi (M. Donduashvili);; Ecole de Santé Publique de Kinshasa, Kinshasa, Democratic Republic of the Congo (E.O. Wemakoy);; Institut National de Recherche Biomédicale, Kinshasa (J.-J. Muyembe);; Laboratoire Veterinaire de Kinshasa, Kinshasa (L. Mulumba);; Universite de Kinshasa, Kinshasa (J. Malekani);; US Centers for Disease Control and Prevention, Kinshasa (J. Kabamba);; US Centers for Disease Control and Prevention, Addis Ababa, Ethiopia (T. Kanter, L.L. Boulanger);; Ethiopian Public Health Institute, Addis Ababa (A. Haile, A. Bekele);; Ethiopian Ministry of Livestock and Fishery Resources, Addis Ababa (M. Bekele);; Addis Ababa Urban Agriculture Bureau, Addis Ababa (K. Tafese)

**Keywords:** zoonoses, public health program implementation, prevention and control, program design, global health security, Republic of Georgia, United States, Democratic Republic of the Congo, Ethiopia

## Abstract

Preventing zoonotic diseases requires coordinated actions by government authorities responsible for human and animal health. Constructing the frameworks needed to foster intersectoral collaboration can be approached in many ways. We highlight 3 examples of approaches to implement zoonotic disease prevention and control programs. The first, rabies control in Ethiopia, was implemented using an umbrella approach: a comprehensive program designed for accelerated impact. The second, a monkeypox program in Democratic Republic of the Congo, was implemented in a stepwise manner, whereby incremental improvements and activities were incorporated into the program. The third approach, a pathogen discovery program, applied in the country of Georgia, was designed to characterize and understand the ecology, epidemiology, and pathogenesis of a new zoonotic pathogen. No one approach is superior, but various factors should be taken into account during design, planning, and implementation.

Rapid detection, response, and control of public health emergencies, including outbreaks of zoonotic diseases, can prevent the international spread of diseases and ensure global health security. In 2014, the Global Health Security Agenda (GHSA; http://www.ghsa.org) was launched to help countries achieve their World Health Organization International Health Regulations (2005) (*1*) obligations of establishing a framework for rapidly detecting, responding to, and controlling infectious disease threats. As of June 2017, a total of 59 countries agreed to contribute to the public health capacity-building efforts of the GHSA. These efforts focus primarily on 11 action packages; specific goals and objectives include preventing zoonotic diseases.

The prevention and control of zoonotic diseases impose a unique, often heavy burden on public health services, particularly in resource-limited settings. Because zoonotic diseases can deeply affect animals and humans, for many zoonotic infections, medical and veterinary health agencies have a large stake in disease surveillance and control activities. Collaboration between agencies is pivotal but takes time, requiring dedicated planning and well-exercised coordination of activities. Achieving this level of collaboration can be daunting in many real-world situations where resource disparities, differences in institutional culture and priorities, disparate legal authorizations, and many other factors can impede development of the formal structures needed to ensure effective implementation of disease prevention and control programs. Field observations and anecdotal reports suggest ongoing risks to human health, to the preservation of wildlife, and, in many cases, to livestock production—the last of which can compound human hardships by negatively affecting livelihoods—in the absence of formal structures that enable intersectoral collaboration.

One-sided disease prevention (enacted either by the human or animal health sector), although well-intentioned, often is inefficient at curtailing the spread of zoonotic infections. For example, in developing countries where canine rabies is still endemic, a rabies prevention program focused primarily on preventing human deaths by increasing access to vaccines for postexposure prophylaxis (PEP), with little or no simultaneous investment in vaccination of dogs, will undoubtedly save lives but is not as cost-effective as investing in mass canine vaccination aimed at eliminating disease from the primary reservoir (*2*). In the absence of efforts to eliminate the source of the virus in dogs, the high costs associated with procurement, distribution, and administration of PEP will persist. Engaging animal and human public health sectors in the implementation of a comprehensive, multisectoral, rabies prevention and control program has a greater and more rapid impact on humans than does using a stand-alone PEP program (*2*). A comprehensive rabies prevention and control program should focus not only on the stockpiling of human rabies vaccine for PEP but also on dog population control, mass canine rabies vaccination, community education, laboratory diagnostic testing, and establishment of joint animal–human rabies surveillance and response systems (*3**,**4*).

Successfully enacting simple measures to promote coordination and multisectoral reporting of suspected disease outbreaks can significantly increase the likelihood of successful disease prevention and control program implementation in resource-limited settings. Jointly training community health workers to build local networks between and among animal and human health providers can empower and enable them to investigate and enact control measures in the context of suspected zoonotic disease outbreaks. A veterinary worker trained to recognize syndromes suggestive of zoonotic disease in humans and given the necessary skills and tools to alert public and animal health authorities on suspected cases can be integral to outbreak detection.

In many circumstances, a precondition for the successful integrated control of zoonotic diseases is the generation of a list of joint zoonotic disease priorities (*5*). Joint multisectoral disease prioritization is important for several reasons. First, a zoonosis of paramount concern to the agricultural or wildlife sector might be of lesser concern to practitioners of human health and vice versa. This lack of awareness between different sectors on how differing disease prevention and control activity affects one another and the overall disease burden reduces buy-in and motivation for allocating resources toward disease prevention and control by the lesser-affected sector. As an example, parapoxvirus infections can confer substantial rates of illness and death on juvenile goats, sheep, and cattle, but human infections are generally mild and self-limited (*6*). In the absence of a specific or new threat, public health authorities might be reluctant to contribute scarce surveillance and laboratory diagnostic resources to building coordinated detection and response capabilities around this infection. The discussion and deliberation of a One Health prioritization process can build consensus and commitment among diverse stakeholders for subsequent implementation activities. On the other hand, decision-makers in animal and human health sectors generally agree on rabies—which exacts a serious toll on humans, companion animals, and livestock alike—as a joint priority. The process of formal prioritization has the additional benefit of encouraging joint review of surveillance systems and data and other health-associated statistics in a deliberative process across ministries. Strengthening surveillance systems, laboratory diagnostic techniques, and response procedures can be applied to other zoonotic diseases with minimal additional investment.

Many possible models of joint program implementation strategies can be aimed at preventing and controlling zoonotic diseases. We highlight 3 distinct approaches that can be considered not only on the basis of resource availability (e.g., human and financial resources) but also on the nature of the disease (Figure 1). The first, rabies in Ethiopia (a Phase 1 GHSA country), illustrates the institution of a comprehensive or “umbrella” approach program for rabies prevention and control, which, although resource intensive, may have a more rapid and transformative effect on disease incidence. The second, monkeypox in the Democratic Republic of the Congo (DRC; a Phase 2 GHSA country), highlights a phased program or stepwise approach to building disease prevention and control capacity based on establishment of a robust foundation of surveillance, followed by augmentation of technical capacities during research activities. The final example is Akhmeta virus in the country of Georgia (a Phase 2 GHSA country). Akhmeta virus, first identified in 2013, causes a zoonosis thought to be derived from wildlife (*7*). The disease first came to light during a cattle-associated outbreak of cutaneous lesions among herders in Georgia. This example demonstrates a pathogen discovery approach that focuses on how discovery of a new zoonosis can stimulate innovation and the motivation for capacity development at the intersection of human, domestic animal, livestock, and wildlife health.

**Figure 1:**
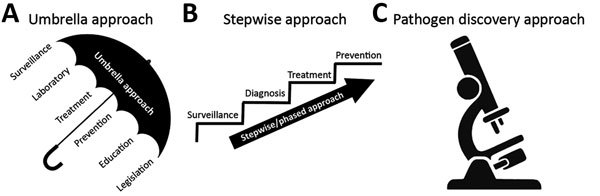
Three program approaches for implementing integrated zoonotic disease detection, prevention, and control programs. A) Comprehensive (umbrella) approach, designed to accelerate collaboration and impact. B) Phased (stepwise) approach in which each step building on prior developed program areas and capacities. C) Pathogen discovery approach, based on the necessity of early intersectoral collaboration to generate knowledge in the context of discovering an emerging zoonotic pathogen, which can subsequently take an umbrella or stepwise approach for program implementation.

## Approaches to Implementing Zoonotic Disease Prevention and Control Programs

### Ethiopia—A Comprehensive (Umbrella) Approach

An example of the use of a comprehensive (umbrella) approach to program implementation is the Rabies Prevention and Control Program implemented in 2015 in Ethiopia. The program involves collaboration and partnership between the Ethiopian Public Health Institute, the Ministry of Livestock and Fisheries, Addis Ababa Urban Agriculture Bureau, and the US Centers for Disease Control and Prevention (CDC) directed toward priority zoonotic diseases identified by the Ethiopian government in September 2015. At the conclusion of the Ethiopia joint zoonotic diseases prioritization workshop, rabies was identified as the priority disease (*8*).

Canine rabies is endemic to Ethiopia; an estimated 105 dog bites/100,000 humans occur per year, and >1.7 deaths/100,000 persons are reported every year (*9*). A prominent element of the GHSA Zoonotic Diseases Prevention and Control Program is a pilot rabies prevention and control program in selected zones in 3 regions and the capital city, Addis Ababa. The rabies program, designed using an umbrella approach, has the potential to impact ≈10.6 million persons. The program was designed to ensure that the basic principles necessary to successfully control canine rabies could be enacted simultaneously in a coordinated manner.

In Ethiopia, the rabies prevention and control program incorporates laboratory-based surveillance; sustained canine mass vaccination programs; increased access to modern cell culture–based human rabies vaccines for PEP; and efforts around education, legislation, and government support. Simultaneous launch of a comprehensive suite of program components is challenging in resource-limited settings. Often for rabies, when resources are limited, vaccines for humans and animal-bite surveillance programs receive the highest priority for funding. Evidence-based program implementation has repeatedly demonstrated that eliminating rabies in dogs is the most cost-effective method to prevent and control the disease (*4*). Although several effective strategies exist for eliminating canine rabies, many countries lack the resources to implement such strategies effectively. The Ethiopia GHSA rabies program benefits from strategic investment of government engagement and intensive technical consultation and assistance, mainly possible because of the large amount of financial resources earmarked toward these efforts. Without such resources, an umbrella approach to program implementation might not have been feasible. International partner resources have supported supplemental staffing of surveillance officers, underwriting training and technical workshops, and procurement of laboratory equipment and consumable supplies. The cross-cutting, comprehensive nature of this program, incorporating elements from 9 of the 11 GHSA action packages (Table), is anticipated not only to save lives with long-term, cost-saving implications but also to serve as a platform for prevention and control of other zoonoses.

**Table:** Capacity-building program areas included in zoonotic disease programs in 3 countries*

GHSA country†	GHSA Action Package												
	Prevent					Detect					Respond		
	AMR	Zoonotic diseases	Biosafety, biosecurity	Immunization		Lab	Surveillance	Reporting	Workforce		EOC	PH law	Medical counter
Ethiopia		√	√	√		√	√	√	√		√	√	
DRC		√	√	√		√	√		√				√
Georgia		√	√			√	√		√				

*AMR, antimicrobial resistance; DRC, Democratic Republic of the Congo; EOC, Emergency Operations Center, GHSA, Global Health Security Agenda; PH, public health. †Ethiopia, GHSA Phase 1; DRC, GHSA Phase 2; Georgia, GHSA Phase 2.

### DRC—A Stepwise Approach

An example of the use of a stepwise approach for zoonotic disease program implementation is the monkeypox detection and prevention program in the Tshuapa Province of DRC, where human disease is endemic. The program began by establishing a strong public health laboratory–based surveillance system, which was used to then gradually introduce additional activities, such as research and applied public health (veterinary and human). Many questions remain about monkeypox virus, including the extent and nature of human-to-human transmission (e.g., whether specific high-risk behaviors are linked to transmission), the precise zoonotic reservoir(s) of the virus, and ecologic determinants of disease incidence (*10*). Evidence suggests that waning vaccine-based immunity conferred by smallpox vaccination might contribute to the increased disease incidence in rural DRC (*11*).

In 2010, CDC partnered with the Kinshasa School of Public Health and the DRC Ministry of Health to strengthen laboratory-based surveillance for monkeypox in the Tshuapa Province. The program provided appropriate specimen collection kits and monkeypox-specific data collection tools; 2 training sessions for ≈60 local animal and human health workers, which emphasized a One Health approach to disease detection and response; the hiring of local staff to periodically reinforce surveillance principles at local public health offices at regular intervals; and diagnostic testing support at the national laboratory (*12*). These efforts increased the number and type of appropriate diagnostic specimens for monkeypox diagnosis submitted to the laboratory for testing (16-fold), the number of cases that were formally investigated (30-fold), and the proportion of laboratory-confirmed monkeypox cases (2.5-fold).

Ministry of Health officials attributed a more rapid recognition and response to the Ebola virus disease outbreak in Lokolia, Tshuapa, in 2014 to the cross-cutting nature and application of the training and surveillance activities provided by the monkeypox program, including reinforcement of key surveillance principles. Persons who had received training under this program ultimately held key leadership roles in the Ebola outbreak response. In addition, because of the multisectoral relationships established through the monkeypox program, Ministry of Agriculture authorities together with the Ministry of Health co-instituted and supported a temporary ban on the sale of animal carcasses suspected to be integral to the transmission of disease until bushmeat consumption could be ruled out as a vehicle for ongoing virus transmission.

The enhancement and reinforcement of a strong surveillance system for monkeypox has resulted in establishment of a foundation on which additional research activities can be added in a stepwise manner. The outcomes and effects have included development of a mechanism to identify geographic locations for longitudinal biologic sampling of wildlife to investigate suspected sylvatic animal species that could be reservoirs for monkeypox virus. Partners from the University of Kinshasa continue to be instrumental in helping design studies, conduct field work, and train young and motivated scientists in DRC. Together with ecologic research activities, epidemiologic research and response activities have been conducted to assess the extent and nature of human-to-human transmission, risk factors for zoonotic introduction of disease in communities, and the extent to which smallpox vaccination might or might not provide long-term protection against disease acquisition >30 years after routine childhood vaccination (*13**,**14*). A partnership with a Congolese educational entity (International Conservation and Education Fund) has proved particularly fruitful by providing evidence-based, locally vetted recommendations for disease prevention, including risks from exposure to wildlife, for tens of thousands of community members.

Overall, these and additional program and research efforts among multiple intersectoral partners greatly increased the capacity to detect and respond to monkeypox disease. Simultaneously, these efforts enabled the gain of critical pieces of scientific knowledge that can be used to protect human lives and develop more efficient evidence-based program implementation options.

### Georgia—An Approach for New Disease Detection Programs

When an emerging zoonotic pathogen is detected, scientists can begin to study its epidemiology, ecology, and pathology using knowledge about closely related organisms as a starting point. Research and surveillance can be initiated simultaneously while in-country partners begin to learn and identify techniques related to sample collection, processing, and diagnostics and build information exchange systems among ministries to facilitate surveillance and response. As part of a joint research and capacity-building program, a coalition of intragovernment partners designed and implemented a research and surveillance program in Georgia using a One Health approach that focused on the new orthopoxvirus, Akhmeta virus, discovered in 2013 (*7*). After this discovery, CDC collaborated with partners at the National Center for Disease Control and Public Health (NCDC) and the National Food Agency in Georgia to initiate a response that focused on examining and collecting data on the epidemiology and characteristics of this virus while simultaneously building laboratory capacity to detect infections in humans and animals through ELISA, PCR, and sequencing diagnostic methods. Coordinated between CDC and the Ministries of Health and Agriculture, the work seeks to expand surveillance for orthopoxviruses while building a knowledge base through epidemiologic, ecologic, molecular, and immunologic research. Partners at NCDC, National Food Agency, and the Laboratory of the Ministry of Agriculture and CDC lead these efforts.

A major ecologic research effort also was initiated through this program to investigate the geographic distribution and seasonal dynamics of Akhmeta virus in potential small mammal reservoirs. At least 700 samples from small mammals have been collected from multiple locations. In addition, studies are under way to establish the burden of disease and identify possible risk factors for human and livestock infections. Samples from humans suspected to have orthopoxvirus infection are sent to NCDC for diagnostic evaluation and positive samples are characterized locally by nucleic acid sequencing and viral isolation.

Although still in its early phases, this collaboration, centered around detecting and investigating a newly identified zoonosis, already has resulted in the discovery of additional instances of human orthopoxvirus infection in Georgia, a greater understanding of other prominent etiologies for cutaneous lesions, isolation of orthopoxvirus from terrestrial rodents, and enhanced collaboration around surveillance and response between the human and veterinary public health sectors. Each innovation has fostered intersectoral collaboration and capacity building across multiple technical areas.

## Conclusions

GHSA is a global initiative that aims to accelerate the progress of participating countries toward achieving their International Health Regulations (2005) obligations of rapidly detecting, responding to, and controlling public health emergencies to enhance global health security. Minimizing the threat posed by zoonotic diseases is one goal of GHSA. The conceptual framework of One Health provides a model on which to build programs to successfully detect, prevent, and control zoonotic diseases (*15*). All 3 suggested approaches for zoonotic disease prevention and control program implementation underscore the importance of strong multisectoral collaboration, engagement, and commitment, essential principles of the One Health framework. Success can be achieved in many ways with any these approaches. A successful program can be designed to be overarching, involving the redesign of entire surveillance and/or laboratory systems to maximize interconnectedness, or it can be constructed to suit a specific context. Programs can focus on known gaps in prevention and control of a particular disease or consider the availability of resources to dictate selection of a specific approach. Optimal approaches will share a foundation of mutual interest across sectors and support a platform for coordinated actions. In the most streamlined form, basic program requirements should comprise surveillance and response activities (human and animal); laboratory diagnostic capacity; data analysis; reporting structures; and the determination of thresholds, triggers, or both that can signal the need for additional action. Recognition of disease in animals may signal the start of an outbreak in humans. Early detection of illness in livestock, companion animals, or wildlife (as was seen in the examples described in Ethiopia, DRC, and Georgia) can alert public health authorities that actions are needed to stem burgeoning risks to humans. Early detection is particularly important where humans heavily depend on livestock production or bushmeat and where peridomestic or domestic animals are prominent.

Establishing systems at the national level with subsequent replication and tiered proliferation to regional and subregional levels (i.e., decentralization) requires constant refinement and modification consistent with local capacities and needs. For a comprehensive program implementation, as described for rabies in Ethiopia, piloting the broad-based integrated system at several distinct locations was determined to be the key first step so that system gaps or inconsistencies could be addressed and costs estimated before nationwide implementation. In DRC, the program for monkeypox detection and control was built step-by-step on a platform of surveillance, with next steps determined by needs and gaps identified through evaluation of data and surveillance performance. Technical capacities were augmented through ongoing program enhancements and research activities. In Georgia, gaps in scientific knowledge about an emerging pathogen drove the initiation of integrated human, livestock, and wildlife disease surveillance and enhancement of laboratory and research capacity.

**Figure 2:**
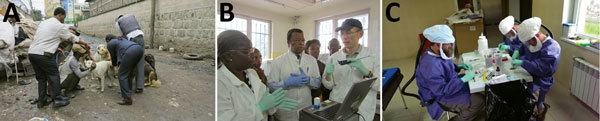
Three program approaches for implementing integrated zoonotic disease detection, prevention, and control programs. A) Comprehensive (umbrella) approach, Ethiopia. Photo credit: Ohio State University. B) Phased (stepwise) approach, Democratic Republic of the Congo. Photo credit: US Centers for Disease Control and Prevention. C) Pathogen discovery approach, country of Georgia. Photo credit: US Centers for Disease Control and Prevention.

The lessons learned through the design and implementation of these programs are continuously derived from persons working at different levels of all contributing institutions. Most significantly, the work should focus on eliminating solely vertical program elements (i.e., those with few or no points of intersection across partner agencies) (Figure 2). Programs should instead work toward integration with existing programs and health systems (both human and animal) when feasible, with points of intersection at all operational levels. Second, continued reinforcement of the key principles and expected effect of the program from the highest levels of participating entities to the lowest is not only conducive to program success but also vital for ongoing material (e.g., financial) and personnel support.

Finally, an indispensable element of GHSA zoonotic disease prevention programs is training of the future workforce. Not just in the animal health sector, where sizable gaps are evident, but also training must be performed to ensure that human public health workers appreciate and know about the importance of veterinary medicine and animal health in controlling zoonotic diseases and that young, university-based scientists have the training and experience necessary to address questions and problems posed by endemic and emerging zoonotic diseases. The training of future public and animal health professionals is a huge component of all 3 programs described in this report.

Achieving the end goal of an effective, fully integrated program for preventing and controlling zoonotic diseases has many possible approaches. The 3 described here differ in their disease-specific context, but all were equally affected by the situation, the resource base, and the initial technical capabilities of the GHSA partner country in which the program was created. The suggested approaches for zoonotic disease program implementation have limitations. Scientific evidence is scant to support 1 approach over another. There is a need for increased zoonotic disease program evaluation and subsequent publication of empirically based recommendations for program design and implementation based on the identified strengths and weaknesses of various approaches. In the interim, national governments and partners can use the approaches we suggest as a guide during the program design phase when they consider suitable approaches for their specific context and settings.
